# Light-Driven Liquid Crystal Circular Dammann Grating Fabricated by a Micro-Patterned Liquid Crystal Polymer Phase Mask

**DOI:** 10.3390/polym9080380

**Published:** 2017-08-21

**Authors:** Xiaoqian Wang, Saibo Wu, Weiqiang Yang, Conglong Yuan, Xiao Li, Zhen Liu, Manchun Tseng, Vladimir G. Chigrinov, Hoising Kwok, Dong Shen, Zhigang Zheng

**Affiliations:** 1 Physics department, East China University of Science and Technology, Shanghai 200237, China; cyber0606@163.com (S.W.); yurkywq@163.com (W.Y.); conghuakun@163.com (C.Y.); lixiaocpf@126.com (X.L.); zhen_liu1@tianma.cn (Z.L.); shen@ecust.edu.cn (D.S.); 2 State Key Laboratory on Advanced Displays and Optoelectronics Technologies (SKL), Hong Kong University of Science and Technology, Hong Kong, China; eemcken@ust.hk (M.T.); eechigr@ust.hk (V.G.C.); eekwok@ecust.edu.cn (H.K.); 3 Liquid Crystal Institute, Kent State University, Kent, OH 44242, USA

**Keywords:** liquid crystals, LC polymer, light-driven, Dammann grating, photoalignment

## Abstract

As one of the diffractive optical elements, circular Dammann grating has shown its excellent versatility in practical applications. The electrically switchable Dammann grating has been extensively investigated; however, the research on the optically tunable circular Dammann grating has received less attention and reports on this subject have been insufficient in the past decade. In this paper, three-order and eight-order binary-phase liquid crystal circular Dammann gratings with two mutually orthogonal photo-induced alignments in every two adjacent alignment domains, fabricated by a micro-patterned liquid crystal polymer phase mask, are proposed to generate annular uniform-intensity patterns in the far field. A simple maskless optical tuning of an eight-order liquid crystal circular Dammann grating is demonstrated by controlling the polarization of an ultraviolet light as well as the energy dose. The proposed liquid crystal circular Dammann gratings with high efficiencies and desirable uniformities exhibit outstanding optical as well as electrical tunabilities, enabling the widespread prospective applications in adaptive photonic chips stimulated flexibly by only light or by the combination of light and electric field.

## 1. Introduction

Recently, liquid crystal (LC) diffractive optical elements (DOEs) [1,2,3,4,5] (e.g., LC gratings, LC lenses, LC airy beam generators, etc.) with attractive features such as excellent electro-optical properties and compact size have inspired the launch of substantive explorations and efficient endeavors in both academia and industry. An LC DOE can be fabricated by several methods, e.g., employing polymer-relief layers [6,7], using patterned electrodes [8,9], patterning the alignment layer, and so on so forth, to achieve a variety of optical behaviors [10,11,12,13]. An LC Dammann grating (DG), as one of the DOEs, is a diffraction grating based on a birefringent material that can uniformly distribute the incident optical energy into the designed diffraction orders [14,15,16,17].

Owing to its high efficiency and outstanding uniformity, a rectangular two-dimensional DG with a binary, multilevel, or continuous phase modulation exhibits excellent performance as an optical array generator. Ge et al. proposed a blue-phase (BP) LC Dammann grating [15] based on two alternating domains with isotropic refractive index and electric-field-induced extraordinary refractive index, which manifested the properties of fast response and polarization independence, but exhibited a crucial flaw of a high driving voltage of ~180 V. Wang et al. reported a binary-phase DG based on a hybrid photo-aligned nematic (HPAN) dual-frequency (DF) LC cell [16] with mutually orthogonal easy axes in every two adjacent alignment domains, which possessed the features of fast switching and low driving voltage. However, the inevitable heat effect of DFLCs under high frequency electric fields could deteriorate their optical properties, which was a big issue in their practical applications [18,19].

Distinct from the rectangular DG, a circular Dammann grating (CDG) generating equal-intensity rings instead of an equal-intensity spot array has been widely used in laser beam shaping, optical image coding [20], collimation testing [21], geometrical measurement [22,23], etc. Zhou et al. fabricated a circular Dammann grating that showed equal-intensity rings in the far field [24]. Later on, Zhao et al. produced a CDG with optimized periodic structure and obtained an annular diffraction pattern with an extremely weak zeroth diffraction order in 2006 [25]. However, the practicability of the abovementioned two CDGs based on the e-beam lithographic technique was restricted by their complicated and challenging fabrication process. In 2011, Luo et al. disclosed a binary-phase CDG by doping azo dye into an LC [17]. The LC CDG with alternating twisted nematic (TN) and homogeneous LC arrangements was fabricated through a multi-exposure photoalignment process, whose maximum zeroth and first/second diffraction orders could be separately achieved by adjusting the analyzer. Nevertheless, such an LC CDG was characterized by a slow response time, polarization dependence, and optical instability due to the azo dye material employed in their experiment.

In this paper, we present optically and electrically tunable binary-phase LC CDGs based on a two-step photoalignment technique, which can experimentally generate equal-intensity rings with a uniformity of up to 0.0765 and an diffraction efficiency approaching 80%. An LC CDG, composed of two indium tin oxide (ITO)-coated substrates with patterned alignment layers, is judiciously designed to satisfy the half-wave condition. Furthermore, the easy axes of the LC molecules in every two adjacent alignment domains are mutually orthogonal, resulting in a π phase shift for the passing light. It is emphasized that the optical tunability and reconfigurability of the LC CDG can be well achieved thanks to the terrific rewritability of the photoalignment material. Once the proposed LC CDG is fabricated by a liquid crystal polymer (LCP)-based amplitude photomask, no mask at all is needed in the dynamic optical tuning process, and the angle between the easy axes of the LC molecules in odd zones and even zones is merely controlled by the polarization of the ultraviolet (UV) light as well as the energy dose. Based on these abovementioned features, the proposed light-driven LC CDG could exploit extensive prospective applications [20,21,22,23,26,27], such as optical data storage, optical coding, communication, etc.

## 2. Materials and Methods

### 2.1. Fundamentals of Circular Dammann Grating

The transmission function *t*(*r*_0_) of the CDG has only two values, 1 and −1, corresponding to the phase value $\varphi_{i}=0\;\;$ and $\varphi_{i}=\pi$. The function *t*(*r*_0_) comprises a series of circle functions and can be written as:

(1) $$\begin{array}{ll} t(r_{0}) & =\sum_{i=1}^{N}\exp(j\phi_{i})[circ(\frac{r_{0}}{a_{i}})-circ(\frac{r_{0}}{a_{i-1}})]\\ & =\sum_{i=1}^{N}{\left(-1\right)}^{i-1}[circ(\frac{r_{0}}{a_{i}})-circ(\frac{r_{0}}{a_{i-1}})] \end{array}$$

where *j* is an imaginary unit, *circ*(*r*_0_) = 1 for *r*_0_ < *a_i_* and *circ*(*r*_0_) = 0 for *r*_0_ > *a_i_*. For *i* = 1, 2, 3 ... *N* (*N* is the overall circle number of the CDG), the set {*a_i_*} is a set of normalized phase transition points with boundary values of *a*_0_ = 0 and *a_N_* = 1. According to Fourier-Bessel transformation, the amplitude of the diffracted electric field at a transverse distance (*r*) to the center of the diffraction pattern can be expressed as:

(2) $$E(r)=\frac{1}{j\lambda z}e^{jkz}\sum_{i=1}^{N}{\left(-1\right)}^{i-1}\{\pi a_{i}{}^{2}[\frac{2J_{1}(ka_{i}r/z)}{ka_{i}r/z}]-\pi a_{i-1}{}^{2}[\frac{2J_{1}(ka_{i-1}r/z)}{ka_{i-1}r/z}]\}$$

where λ is the wavelength, *k* is the wave number, and *z* is the distance from the diffractive optical element to the observation screen.

Hence, the light intensity at *r* can be calculated by taking the square of the absolute value of the electric amplitude:

(3) $$I(r)={\left|E(r)\right|}^{2}$$

The diffraction efficiency of the LC CDG is defined as:

(4) $$\eta =\sum_{i=0}^{M}I_{i}/I_{total}$$

where *I_i_* is the intensity of the *i*th order and *I_total_* is the intensity of the incident light. Furthermore, its uniformity is defined as:

(5) $$u=\sum_{i=0}^{M}{\left(I_{i}-I_{av}\right)}^{2}/\sum_{i=0}^{M}I_{i}$$

where *I_av_* is the average intensity of all of the diffraction orders.

### 2.2. Method of Fabricating Liquid Crystal Circular Dammann Gratings

**Figure 1:**
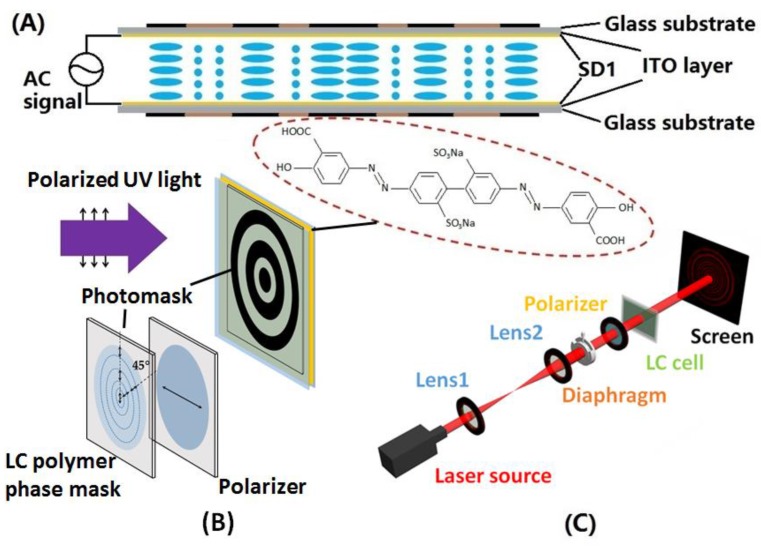
The fabrication of the proposed liquid crystal (LC) circular Dammann grating (CDG) and the experimental setup for measurement. (**A**) Schematic side view of the configuration of the LC CDG driven by an alternating current (AC) signal. (**B**) The photoalignment process with the molecular structure of SD1 shown in the red dotted oval. (**C**) The experimental setup for measuring the optical properties of the LC CDG.

By optimizing the normalized radius parameters of the CDG, the annular optical energy distribution with desirable uniformity and high efficiency can be realized. Figure 1A shows the schematic side view of the configuration of the LC CDG, which comprises two ITO-coated glass substrates with photoalignment layers. The black and brown segments above and beneath the glass substrates represent the odd zones and the even zones of the CDG, respectively. The designed arrangements of LC molecules in odd and even zones, which are oriented orthogonal to each other, endow the LC CDG with polarization independence [28].

A sulfonic azo dye, the photoalignment material SD1 (Dai-Nippon Ink and Chemicals, Tokyo, Japan), whose molecular structure is shown in the red dotted oval in Figure 1B, is treated to form an optically active alignment layer [29]. When the photosensitive SD1 layer is exposed to a linearly polarized UV light with the wavelength of ~365 nm, the energy absorbed by SD1 molecules is proportional to cos^2^ *θ*, where *θ* is the angle between the azo dye chromophore and the polarization plane of the UV light, resulting in the in-plane rotation of the molecules, eventually orienting perpendicular to the polarization plane of the UV light after a sufficient energy dose [30]. Moreover, the SD1 layer provides almost zero pre-tilt angle and high anchoring energy [31].

To fabricate the desired LC CDG, two substrates with conductive layers were coated with SD1 (0.5 wt % in *N*,*N*-dimethylformamide) and exposed under a linearly polarized UV light (*I* = 5.5 mW/cm^2^) with an energy dose of 5 J/cm^2^ to make the initial alignment. Then, the substrates were assembled to form an LC cell with 5 μm spacers. The empty LC cell was irradiated at normal incidence by a linearly polarized UV beam with its polarization plane orthogonal to the initial alignment direction through a designed amplitude photomask, which was put in close contact with the prepared cell. The amplitude photomask consisted of a micro-patterned photo-polymerized LC (30 wt % UCL017A in propylene glycol monomethyl ether acetate (PGMEA), from DIC, Tokyo, Japan) phase mask (space-variance half-wave plate) with the alignment directions in odd and even zones making an angle of 45° to each other and a polarizer with its transmission axis orthogonal to the alignment direction in odd zones of the phase mask (parallel to the initial alignment on the prepared substrate) (see Figure 1B). The linearly polarized UV light passing through odd zones of the LCP phase mask was blocked by the polarizer, and the polarization of that passing through even zones was rotated 90° and then further purified by the polarizer in order to make the polarization of the outgoing light strictly parallel to the initial alignment on the substrate. Thus, the SD1 molecules in even zones were realigned with their easy axes orthogonal to the initial orientation of those in odd zones. The advantages of the LCP-based amplitude photomask over conventional metallic ones produced by photolithography include easy fabrication, economical manufacture cost, accessible reproduction via polarization holography technology [3], etc. Thereafter, a commercial nematic liquid crystal SLC1717 (from SliChem, Shijiazhuang, China) with the birefringence ∆*n* = *n_e_* − *n_o_* ≈ 0.201 at 633 nm was infiltrated into the cell by capillary action. Hence, the LC cell approximately satisfied the half-wave condition, and the deviation of the cell gap to the designed value was about 0.3 μm.

### 2.3. Characterization

The fabricated LC CDG (see Figure 1C) was then put in the experimental setup for measuring its optical properties, i.e., the far-field diffraction pattern, the diffraction efficiency, and the uniformity. The He-Ne laser (*λ* = 632.8 nm, *I* = 8.3 mW/cm^2^) was used to characterize the LC CDG. The laser beam was expanded by two separated lenses, and then the propagating beam impinged on the LC CDG through an adjustable diaphragm, projecting the far-field diffraction pattern on the screen placed at a distance of ~200 cm away from the LC cell.

### 2.4. Method of Optically Tuning a Liquid Crystal Circular Dammann Grating

To study the optical tunability of the LC CDG, the polarization of the UV light was rotated to a certain angle *α* with respect to the alignment direction in the even zones. Meanwhile, a saturated electric field was applied across the LC cell during the UV irradiation in order to avoid the generation of the TN LC arrangements, which might disturb the photo-patterning process. The dynamic variation of the angle *β* between the alignment directions in odd and even zones might depend on many factors, e.g., the polarization angle of the UV light, the energy dose, the elastic parameters of the used LC, the cell gap, etc. In the experiment, the polarization angle *α* and the energy dose in relationship with the dynamic variation of the angle *β* were investigated, and the evolution of the far-field diffraction pattern was also observed.

## 3. Results and Discussion

### 3.1. Efficiencies and Uniformities of Liquid Crystal Circular Dammann Gratings

A three-order LC CDG with the aperture size of ~4 mm and an eight-order LC CDG with almost the same aperture size were fabricated, whose alignment direction in odd zones was at 90° with respect to that in the even zones. The parameters of the LC CDGs are listed in Table 1. The diffraction efficiencies of the three-order and the eight-order LC CDGs were 78.6% and 76.4%, respectively. Due to the 0.3-μm cell gap deviation, the LC CDGs did not exactly satisfy the half-wave condition, causing an undesired high intensity of the zeroth diffraction orders and deteriorating the uniformities, which were measured to be 0.0765 for the three-order LC CDG and 0.042 for the eight-order one. Ignoring the influence of the zeroth-orders, the uniformities of the non-zeroth diffraction orders for the three-order and the eight-order LC CDGs decreased down to 0.0009 and 0.0014, respectively.

### 3.2. Microstructures and Far-Field Light Intensity Distributions

**Figure 2:**
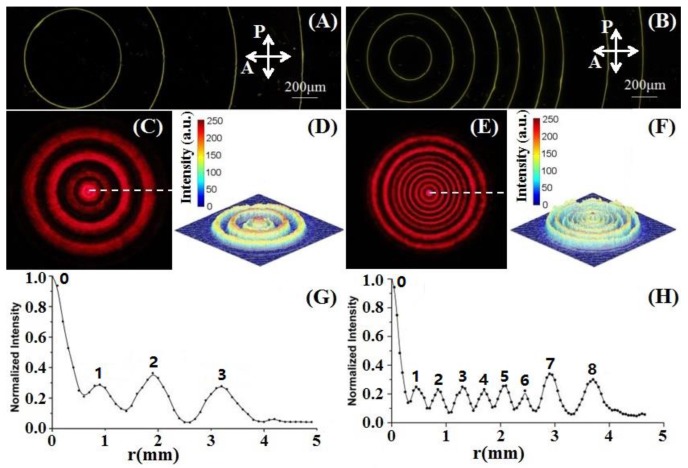
Micrographs of two LC CDGs and their far-field light intensity distributions. (**A**,**B**) Micrographs of the three-order and the eight-order LC CDGs, respectively. Line scales represent 200 μm. (**C**,**E**) Far-field diffraction patterns for the three-order and the eight-order LC CDGs, respectively. (**D**,**F**) Corresponding light intensity profiles for the two LC CDGs. (**G**,**H**) Light intensity distributions along the white dashed lines in (C,E), respectively.

As shown in Figure 2A,B, the micrographs of the three-order and the eight-order LC CDGs were captured under the polarizing optical microscope (POM) with two crossed polarizers. The bright lines in the micrographs are the boundaries between two different alignment domains, which are also regarded as defects. Then, a Canon EOS 70D digital camera was used to capture the images of the far-field diffraction patterns of the LC CDGs (see Figure 2C,E). Analyzed by a self-written MATLAB code for image processing, the corresponding light intensity profiles for the three-order and the eight-order LC CDGs are shown in Figure 2D,F, respectively. Due to the light saturation of the zeroth-orders in the captured images, as shown in Figure 2C,E, the light intensities depicted in Figure 2D,F also became saturated. To obtain the precise light intensity distributions around each diffraction order, a photo-detector was put behind a 0.4-mm diameter pinhole that was placed at a distance of ~200 cm from the LC cell to record the light intensities at transversely uniformly-spaced spots. Thus, the corresponding light intensity distributions along the white dashed lines in Figure 2C,E are illustrated in Figure 2G,H, respectively. In these two figures, the light intensities of zeroth-orders are much higher than those of the rest of the diffraction orders, and the uniformities of the non-zeroth diffraction orders seem quite acceptable, in accordance with the theoretical calculations on the cell gap deviation [24], which can be significantly improved by selecting more proper spacers.

### 3.3. Optical Tunability of a Liquid Crystal Circular Dammann Grating

**Figure 3:**
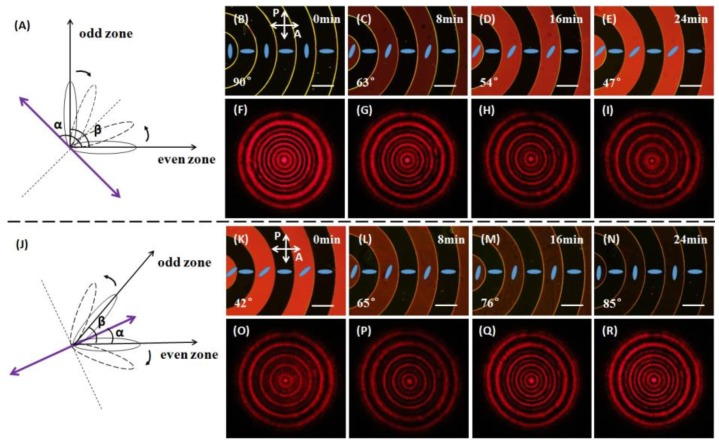
Schematic diagrams of the optical tuning process, and micrographs under polarizing optical microscope (POM) with two crossed polarizers and diffraction patterns of the LC CDG obtained with the polarization of the incident light along the orientation of the LC molecules in the even zones. Line scales represent 200 μm. (**A**) Optical tuning process for reducing the angle *β*. (**B**–**E**) Micrographs with the angle *β* decreasing from 90° to 47° as the exposure time increases. (**F**–**I**) The corresponding far-field diffraction patterns with the even diffraction orders, except for the eighth order, fading away. (**J**) Optical tuning process for enlarging the angle *β*. (**K**–**N**) Micrographs with the angle *β* increasing from 42° to 85° as the exposure time increases. (**O**–**R**) The corresponding far-field diffraction patterns with the even diffraction orders, except for the eighth order, fading in.

Fundamentally, the optical tunability of an LC CDG is based on controlling the aforementioned angle *β* by means of a photoalignment technique. As shown in Figure 3A, an LC CDG with the angle *β* of 90° is exposed to the UV light, of which the polarization (purple double-headed arrow) makes an angle *α* of 90° + *β*/2 with respect to the alignment direction in the even zones. The straight dashed line indicates the inclined alignment direction which is perpendicular to the polarization of the UV light. SD1 molecules in either odd zones or even zones tend to reorient orthogonally to the polarization of the UV light, therefore the angle *β* decreases with the increasing of the energy dose (or exposure time). The LC CDG with the angle *β* from 90° to 42° was achieved, and four representative states were selected to present in this paper. The corresponding micrographs under the POM with two crossed polarizers were observed (see Figure 3B–E), and the far-field diffraction patterns (see Figure 3F–I) were captured. The blue rods (see Figure 3B–E) indicate the orientation of the LC molecules in each domain. Note that the LC CDG is polarization independent only when the angle *β* is equal to 90°, thus the polarization of the probe beam has to be fixed along the alignment direction in the even zones. As shown in the upper half of Figure 3, it took 24 min for the exposed light to change the angle *β* from 90° to 47°; accordingly, except for the outmost ring (the eighth diffraction order), the even diffraction orders gradually faded away, but the odd diffraction orders remained slightly changed. The angle *β* could be further reduced by increasing the energy dose. However, the odd diffraction orders would start to get blurry when *β* is less than 45° and the diffracted ring-pattern would vanish with a very small angle *β*. Therefore, the results of the angle *β* less than 40° do not need to be presented in this paper. Reversely, the angle *β* of the same LC cell was enlarged by rotating the polarization angle *α* to *β*/2, as shown in Figure 3J. The selected micrographs under the POM are illustrated in Figure 3K–N, and the corresponding diffraction patterns are shown in Figure 3O–R. As shown in the lower half of Figure 3, it took 24 min for the exposed light to change the angle *β* from 42° to 85°; correspondingly, the even diffraction orders except for the outmost ring gradually faded in. In the whole optical tuning process, the shape of the boundaries in the micrographs (see Figure 3B–E and K–N) remained unchanged, and the orientation of the LC molecules in each domain kept quite uniform as the LC cell underwent UV irradiation. Therefore, it was evident that the LC molecules with the same orientation would tend to rotate synchronously under the linearly polarized UV light, thus providing the photoalignment material SD1 with an excellent rewritability. Moreover, the optical tuning time can be dramatically reduced to sub-second periods by using a high-power blue laser [32], making it possible for the proposed LC CDG to find applications in abundant modern devices.

### 3.4. Electrical Tunability of a Liquid Crystal Circular Dammann Grating

Due to the intrinsic property of the liquid crystal, the electrical tunability of the LC CDG can be facilely achieved through the electrically induced phase modulation. An investigation of the electro-optical properties of the LC CDG was carried out as well. The diffraction efficiency and the uniformity could be electrically tuned within 1.5 cycles. More details are described in the Supplementary Materials.

## 4. Conclusions

In conclusion, we have proposed three-order and eight-order light-driven LC CDGs that can generate annular uniform-intensity patterns in the far field. The diffraction efficiencies and the uniformities of the LC CDGs reached up to 78.6% and 0.0765, respectively. The optical tuning of the even diffraction orders (except the eighth order) of the eight-order LC CDG was achieved by controlling the polarization of an exposed UV light as well as the energy dose. Due to the maskless optical manipulation, the tuning process was quite simple and convenient; in addition, fast optical tuning could be realized by using a high-power blue laser. Moreover, the electrical tunability of the LC CDG was also investigated. The proposed light-driven LC CDG exhibited outstanding optical as well as electrical tunabilities, thereby promoting promising and exciting applications in advanced stimuli-directed photonic integrated devices.

## Figures and Tables

**Table 1:** Parameters and optical performances of the binary-phase LC CDGs.

Order Number	Normalized Radius *a_i_*	Efficiency	Uniformity
3	0.2178, 0.3922, 0.7194	78.6%	0.0765
8	0.0962, 0.1936, 0.2894, 0.3904, 0.4854, 0.5966, 0.6844, 0.8856	76.4%	0.0420

## Supplementary Materials

The following are available online at www.mdpi.com/2073-4360/9/8/380/s1, Figure S1: Evolution of the far-field diffraction pattern with the increasing voltage. The phase retardation decreases from 3.175π to 2π (corresponding to (a–d)), then decreases further to π (corresponding to (e–g)), and finally diminishes to 0 (corresponding to (h–j)).
