# Supplementary material for: Light-Driven Liquid Crystal Circular Dammann Grating Fabricated by a Micro-Patterned Liquid Crystal Polymer Phase Mask
Source: Polymers (Basel). 2017 Aug 21;9(8):380. doi: 10.3390/polym9080380 (PMC6418637; doi:10.3390/polym9080380)
Supplement: Supplementary file 1 [file polymers-09-00380-s001.pdf]

polymers

MDPI

Supplementary Materials: Light-Driven Liquid Crystal Circular Dammann Grating Fabricated by a Micro-Patterned Liquid Crystal Polymer Phase Mask

Xiaoqian Wang [1,†], Saibo Wu [1,†], Weiqiang Yang [1], Conglong Yuan [1], Xiao Li [1], Zhen Liu [1], Manchun Tseng [2], Vladimir G. Chigrinov [2], Hoising Kwok [2], Dong Shen [1] and Zhigang Zheng [1,3,*]

1 Physics department, East China University of Science and Technology, Shanghai 200237, China; xqwang@ecust.edu.cn (X.W.); cyber0606@163.com (S.W.); yurkywq@163.com (W.Y.); conghuakun@163.com (C.Y.); lixiaocpf@126.com (X.L.); zhen_liu1@tianma.cn (Z.L.); shen@ecust.edu.cn (D.S.)
2 State Key Laboratory on Advanced Displays and Optoelectronics Technologies (SKL), Hong Kong University of Science and Technology, Hong Kong, China; eemcken@ust.hk (M.T.); eechigr@ust.hk (V.G.C.); eekwok@ecust.edu.cn (H.-S.K.)
3 Liquid Crystal Institute, Kent State University, Kent, OH 44242, USA
* Correspondence: zgzheng@ecust.edu.cn; Tel.: +86-21-6425-3514
† These authors contributed equally to this work.

In our experiment, a rectangular wave alternating current (AC) of 1 kHz was applied across the liquid crystal (LC) cell to investigate the electrical tunability of the eight-order LC circular Dammann grating (CDG) with the angle β of 90°. By modulating the amplitude of the AC signal, the light intensity distribution of the corresponding diffraction pattern changed, as shown in Figure S1. The phase modulation of the LC cell can be electrically tuned within the range from 3.175π to 0, going through half-wave condition twice, i.e., 3π and π. From Figures S1a–d, the phase retardation of the LC cell decreased from 3.175π to 2π. The diffraction efficiency reached up to the maximum at the phase retardation of 3π and then decreased to the minimum, as the voltage increased from 0 V to 1.45 V. The diffraction pattern showed clear equal-intensity rings (see Figure S1a) in the absence of voltage. As the voltage rose up to 1.14 V, the even diffraction orders except the outmost ring (the eighth diffraction order) gradually faded away. When the voltage reached up to 1.27 V, the odd diffraction orders got blurry, and all diffraction orders disappeared at 1.45 V. As the voltage further increased to 1.65 V, the odd diffraction orders appeared indistinctly. Meanwhile, the diffraction efficiency increased till the maximum at 2 V, as the phase retardation decreased from 2π to π (corresponding to (d–g)). Eventually, the diffraction efficiency went down from the maximum to the minimum with the phase retardation vanishing.

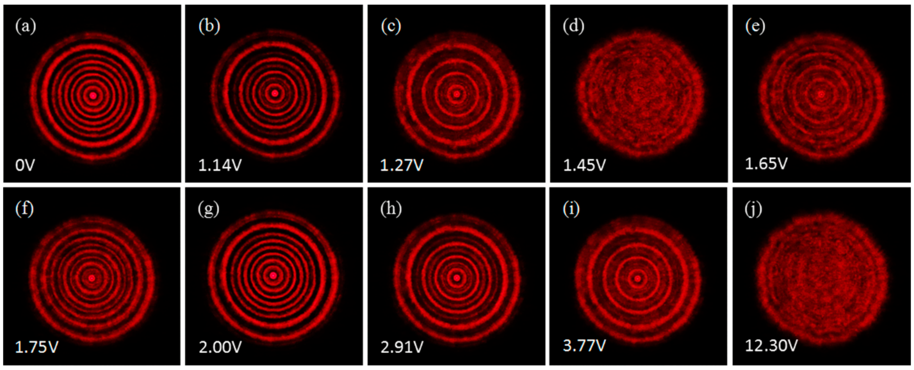

Figure S1. Evolution of the far-field diffraction pattern with the increasing voltage. The phase retardation decreases from 3.175π to 2π (corresponding to (**a**–**d**)), then decreases further to π (corresponding to (**e**–**g**)), and finally diminishes to 0 (corresponding to (**h**–**j**)).
